# Group psychedelic-assisted therapy for mental health conditions: a mixed-methods systematic review

**DOI:** 10.3389/fpubh.2026.1788352

**Published:** 2026-10-06

**Authors:** Vivian W. L. Tsang, Faizan Bhatia, Anne Ferrey, Jamie Hartmann-Boyce

**Affiliations:** 1 Department of Psychiatry, Faculty of Medicine, University of British Columbia, Vancouver, BC, Canada; 2 Evidence-based healthcare, Department of Continuing Education, University of Oxford, Oxford, United Kingdom; 3 Nuffield Department of Primary Care Health Sciences, University of Oxford, Oxford, United Kingdom; 4 School of Public Health and Health Sciences, University of Massachusetts Amherst, Amherst, MA, United States

**Keywords:** addictions, group therapy, mental health, psychedelic-assisted therapy, trauma

## Abstract

**Background:**

The group therapy model has been recently favored as a means of conducting psychedelic assisted therapies for mental health disorders. However, there is a paucity of literature showing strengths or drawbacks of this approach and comparisons of safety and efficacy to individual therapy. This systematic review aims to address this gap in literature.

**Methods:**

We included original research of group psychedelic therapy programs that were peer reviewed and published in English or where a translation was available or possible. Participants must be 18 or older and have a psychiatric diagnosis. We searched for MEDLINE, Scopus, and APA PsychInfo until January 1 2026. Screening, data extraction, and appraisal using the Mixed Methods Appraisal Tool (MMAT) were conducted by two independent researchers. A parallel-results convergent synthesis design was employed consisting of independent syntheses of qualitative and quantitative evidence and an interpretation of the results in the discussion.

**Results:**

We included 30 papers representing 22 unique group therapy protocols. Demographics were inconsistently reported: sex or gender was reported in 18 papers, race/ethnicity in eight papers. Fifteen papers reported pre–post outcomes using validated screening tools for mental health, functioning, substance use, or wellbeing. Using a segregated methodologies approach, quantitative and qualitative findings were broadly complementary. Quantitative results showed overall decreases in validated scale outcomes reflecting improvement in depression, anxiety, trauma symptoms, functioning, and other psychiatric outcomes. Qualitative results described improvements in mindfulness, emotional processing, psychological flexibility, meaning-making, and social connectedness supported by the group format.

**Discussion:**

Despite emerging mechanistic evidence for direct effects of psychedelic molecules on the brain, only a minority of studies used comparison groups, and only one was a modern randomized controlled trial. Group PaT may not only be pragmatically beneficial for feasibility and cost-effectiveness, but may also contribute to therapeutic outcomes through peer support, normalization, belonging, and integration. Limitations of this systematic review include substantial heterogeneity in molecules, populations, protocols, outcome measures, and follow-up duration, as well as overlapping cohorts and few studies with group components throughout the entire course of treatment.

**Systematic review registration:**

PROSPERO: https://www.crd.york.ac.uk/PROSPERO/view/CRD42024491478, identifier: CRD42024492648.

## Introduction

In recent years, there has been a renewed interest in the use of psychedelics to treat a variety of mental health conditions and substance use disorders as an alternative or adjunctive treatment to traditional psychiatric interventions (1). This is especially considered when other methods have failed to achieve remission (2). Psychedelic-assisted Therapy (PaT) is described as the use of psychedelics as an addition to therapy modalities (2, 3). PaT shows significant promise in the treatment of several clinically challenging conditions, including depression, anxiety, and posttraumatic stress disorder (PTSD), among others (4–6).

Currently, most PaT studies largely focus on individual one-on-one treatment due to regulatory barriers and feasibility issues with group PaT (7). However, there is growing evidence for the utility of group therapy modalities (8). Group PaT has been largely demonstrated to be safe and feasible (9, 10). Group PaT has the potential to offer a pragmatic cost-effective alternative while potentially providing additional intrinsic community support and an improved sense of connection to others (11).

Some research has been done in the dawn of psychedelic medicine from the 1950s−1990s and a systematic review of 12 studies by Trope et al. (12) documents the findings of PaT used in group settings. Although minimal or no criteria were applied in many of these studies, the general population involved in the review tended to be patients with varied psychiatric diagnoses—ranging from self-identified to formal clinical investigation. Since then, there has been an influx of recent studies published on PaT and the purpose of this systematic review is to present an update to Trope et al.'s paper using more rigorous DSM5 screening criteria to involve studies that specifically use PaT to address mental health disorders.

Given the newfound consideration of involving psychedelics in mental health treatment and care, the aim of this systematic review is to evaluate the potential benefits and harms of group PaT in patients with a psychiatric diagnosis as well as its effectiveness.

## Methods

The systematic review was conducted to gather information about the effects of group PaT. The Reporting Items for Systematic Reviews and Meta-Analyses (PRISMA) guidelines were used to provide structure for the review reporting. This has been included as a Supplementary Appendix S1. PROSPERO registration for this review is: CRD42024492648.

### Inclusion and exclusion criteria

Inclusion criteria consisted of study types including randomized controlled trials, other types of clinical trials, qualitative or quantitative prospective or retrospective comparative cross sectional, case-controlled, or cohort studies, case reports, and case series that were peer reviewed and published. Studies in English or that have an English translation or that were able to be translated using Google translate were included. Studies that included adult humans (18 years or older) who have a psychiatric diagnosis as defined by study authors or according to clinical criteria (DSM-V) were included. Interventions of both administration of any psychedelic medicines or accepted psychedelic-like substances such as ketamine and demonstration of a group (*n* > 2) therapy component were included. Therapy was defined as any mention of a therapist providing treatment or clear description of types and/or description of group therapy modality utilized. Given the broad perspective for interventions of interest, the comparison that was included as a secondary objective is any mention of comparison to individual PaT. However, the search strategy was kept broad in order to capture all studies on group PaT and then further full text screening accounted for the comparison criteria in our secondary objective. Endpoints important for impacts on mental and physical wellness were of primary interest. Studies were selected for inclusion from all published studies in databases. There were no restraints on follow-up time. There were no restrictions by type of setting. Exclusion criteria included any studies involving humans without mental health conditions, studies that did not administer psychedelic medicines and/or did not clearly outline a group (*n* > 2) therapy component in the methods section of the study protocol, studies that did not report on any of the primary outcomes of interest (which include any impact on mental, physical wellness or social connectedness). Reviews, commentaries, editorials, letters to the editor, conference proceedings, and abstracts were excluded.

### Search strategy

Literature search strategies were developed using medical subject headings (MeSH) and text words related to group PaT. We searched MEDLINE (OVID interface, 1948 onwards), Scopus (includes EMBASE 1980 onwards), and APA PsychInfo until January 1 2026. Electronic database searches were supplemented by mining gray literature sources from Google Scholar (keywords from the search strategy below was typed into Google Scholar and studies that are of interest based on available title and abstract was added to the full text screening stage) and handmining reference lists from identified studies and relevant reviews.

To enhance reproducibility given the dynamic nature of Google Scholar search results, searches were conducted in a cleared browser (incognito mode) without personalization, and results were sorted by relevance. The first 200 results were screened, consistent with prior methodological recommendations for gray literature searching. Titles and abstracts (where available) were screened for relevance, and potentially eligible studies were retrieved for full-text review.

Studies identified through Google Scholar were screened using predefined inclusion criteria and exclusion criteria. All records identified through Google Scholar were imported into Covidence and underwent duplicate removal and independent screening by two reviewers, consistent with the primary screening process.

### Data management

Literature search results were uploaded to COVIDENCE, an internet based software program that facilitates collaboration among reviewers during the study selection process. Two independent reviewers completed title and abstract screening and again for full text screening based on the inclusion and exclusion criteria. Any inconsistencies were resolved by discussion before a third reviewer was involved.

Although ketamine is pharmacologically distinct from classic serotonergic psychedelics [e.g., psilocybin, lysergic acid diethylamide (LSD), 3,4-methylenedioxymethamphetamine (MDMA)], it was included in this review due to its established use within similar treatment frameworks, particularly in structured therapeutic settings incorporating preparation, dosing, and integration phases. Ketamine produces acute changes to consciousness, including dissociation, perceptual changes, and shifts in self-referential processing, which overlap with psychedelic experiences.

Furthermore, KAT has been operationalized in both individual and group-based treatment models, making it directly relevant to our research question. Given its increasing clinical use and inclusion in emerging PaT paradigms, the inclusion of ketamine was imperative in ensuring validity and applicability of findings to current practice.

### Data extraction

A pilot data extraction form was developed before commencing data extraction. This was based on key PICO characteristics as well as the objectives of the proposed review (Table 1). Two independent reviewers extracted data to a prepared excel spreadsheet from the full text screening. Different data types were extracted into the pilot extraction form due to the heterogeneity of studies included in the review. Two independent reviewers subsequently performed data analysis of the included studies, with documentation on demographics, methods, and results compiled into a single, standardized table in Microsoft Excel. Any inconsistencies were resolved through discussion and if needed through referral to a third independent reviewer. EndNote was used for reference management when writing the paper.

**Table 1:** PICOS table.

Attribute	Details
Population	Adults (≥18 years) with a diagnosed psychiatric condition (e.g., depression, anxiety disorders, PTSD, substance use disorders), as defined by study authors or DSM-based criteria
Intervention	Group-based PaT, defined as administration of a psychedelic substance (e.g., psilocybin, ketamine, LSD, MDMA) combined with a structured group therapy component (*n* > 2 participants)
Comparator	Individual PaT; (i) group therapy without psychedelic administration; (ii) placebo or alternative pharmacologic intervention; or (iii) no comparator (single-arm studies included)
Outcomes	Primary outcomes: changes in validated psychiatric symptom scales (e.g., PHQ-9, GAD-7, PCL-5, HAM-D, MADRS) Secondary outcomes: (i) general psychological wellbeing/function (e.g., B-IPF, VAS), (ii) social connectedness/interpersonal functioning (qualitative or quantitative), (iii) substance use outcomes, (iv) safety/adverse events
Study design	Randomized controlled trials, non-randomized trials, cohort studies, case-control studies, cross-sectional studies, qualitative studies, mixed-methods studies, case series, and case reports (peer-reviewed)

Outcomes were categorized *a priori* into primary and secondary domains to improve conceptual clarity and comparability across heterogeneous study designs. Primary outcomes were defined as changes in psychiatric symptom severity, measured using validated clinical instruments, including but not limited to Depression: PHQ-9, HAM-D, MADRS, Anxiety: GAD-7, HAM-A and PTSD: PCL-5.

Secondary outcomes include general psychological wellbeing (defined as subjective wellbeing and results of BIPF, Sheehan Disability Scale, or VAS and the EQ-5D), social connectedness (defined as perceived interpersonal relationship changes, social engagement), substance use outcomes, and safety and adverse events. There were no restrictions to follow-up windows.

### Data synthesis

Studies were first grouped based on research methods (quantitative, qualitative, or mixed-methods) and data collection methods. Results and appropriate synthesis methods were selected based on the studies identified for inclusion. Given that the majority of included studies employed uncontrolled pre–post designs, effect estimates were interpreted as associational rather than causal.

A meta-analysis was not possible due to heterogeneity of studies. A systematic narrative synthesis was conducted with information presented in the text and tables to summarize and explain the characteristics and findings of the included studies. The narrative synthesis explored the relationship and findings both within and between the included studies, in line with the guidance from PRISMA-P. The Synthesis without Meta-analysis guideline (SWiM) was used as a guide for reporting. These included grouping studies for synthesis based on outcome domain, substance class, and study design, describing the synthesis methods, criteria used to prioritize results for summary and synthesis, investigation of heterogeneity in reported effects, certainty of evidence, data presentation methods, reporting results, and limitations of the synthesis (13). Where sufficient data were reported, standardized effect measures (e.g., mean change scores, percentage change, or reported effect sizes) were extracted. Confidence intervals were reported when available; however, most studies did not provide sufficient data for consistent calculation of standardized effect sizes. Qualitative findings from included studies were synthesized using a descriptive narrative approach. This approach was selected to summarize and organize reported qualitative and mixed-methods findings across heterogeneous studies, without undertaking formal thematic synthesis or theory generation.

A parallel-results convergent synthesis design was employed consisting of independent syntheses of qualitative and quantitative evidence and an interpretation of the results in the discussion (14). This was done using a segregated mixed-methods analysis. According to Sandelowski et al.,

“Segregated methodologies' maintain a clear distinction between quantitative and qualitative evidence and require individual synthesis to be conducted prior to the final ‘mixed-methods' synthesis. The findings or evidence fall into categories: the quantitative and qualitative findings may either support each other (confirmation) or contradict each other (refutation); or they may simply add to each other (complementary).” (15)

The same quality assessment tool Mixed Methods Appraisal Tool (MMAT) was used for all studies in order to maximize homogeneity and comparability. Each study was evaluated by two independent reviewers, with inconsistencies and conflicts resolved through discussion.

## Results

In total, 1192 references were imported into Covidence, 258 duplicates were removed automatically and 18 duplicates removed manually. This resulted in 916 studies screened and 70 full text studies assessed for eligibility. This resulted in 30 papers included in the final data extraction (Figure 1).

**Figure 1:**
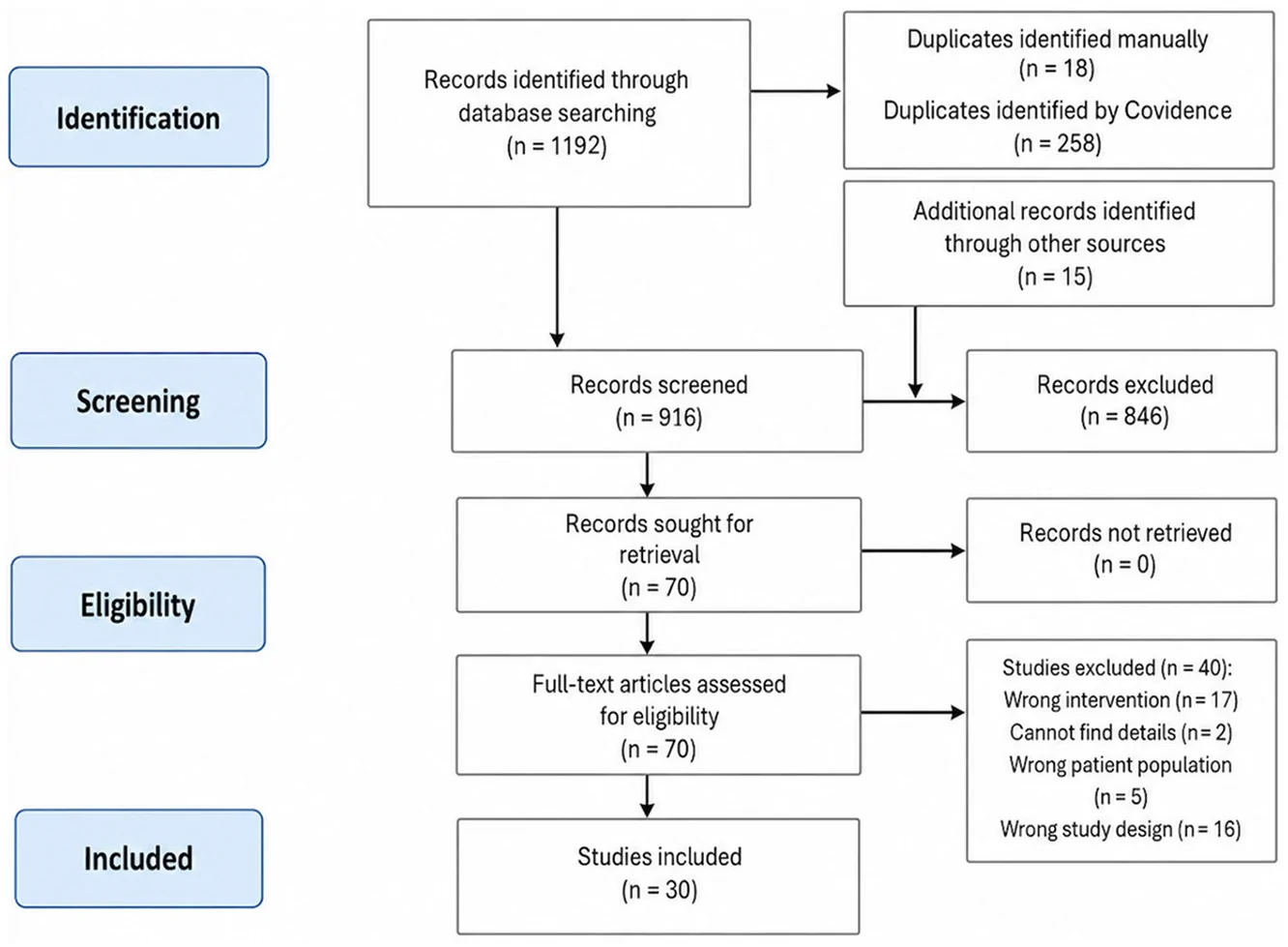
PRISMA flow diagram.

Two papers, Lewis et al. (14, 16) were found to be publications by the same research group using the same therapy protocol and same participants so the outcomes from these studies were combined where relevant. Similarly, five papers [Dames et al. (11), Tsang et al. (15, 17–19)] were also found to be publications by the same research group using the same protocol and patients and were combined. Agrawal et al. (20) and Beaussant et al. (21) published a quantitative and qualitative report, respectively, of their phase 2 open label trial and thus these were regarded as the same study. Two studies Flynn et al. (22, 23) were also combined. This resulted in 22 unique group therapy protocols for analysis. Because several reports described the same underlying participant samples, there were 804 unique individuals captured in the included studies (Table 2).

**Table 2:** Participant mapping.

Cohort/protocol ID	Publications mapped to cohort	Substance	Primary diagnosis/population	Paper-level *N* reported	Unique *N* counted	How handled
Anderson/Agin-Liebes HIV survivor psilocybin group therapy cohort	Agin-Liebes et al. (37)	Psilocybin	Older gay-identified men with HIV-related demoralization/trauma symptoms	9	9	Qualitative subsample counted once
Sunstone/Aquilino cancer MDD psilocybin trial	Agrawal et al. (20, 31); Beaussant et al. (21)	Psilocybin	Cancer + major depressive disorder	30; 28; 28	30	Agrawal, 2023 treated as parent cohort; follow-up and qualitative reports not double-counted
Cheek/Osmond/Sarett alcoholism LSD project	Cheek et al. (24)	LSD	Alcohol use disorder	28 LSD-treated; 15 wives in companion report	28	Sarett treated as collateral spouse-report paper, not an additional treated cohort
Chwelos LSD alcoholism cohort	Chwelos et al. (33)	LSD	Alcohol use disorder	40	40	Distinct cohort
Roots to thrive ketamine-assisted therapy program	Dames et al. (11); Tsang et al. (17) pilot; Tsang et al. (15) safety/tolerability; Tsang et al. (19); Tsang et al. (18)	Ketamine	Healthcare providers, first responders, firefighters, community mental health cohorts	94; 57/38 ketamine; 128; 6; 8	128	Treated as overlapping RTT clinical program; Dames 2025 cumulative count should not be summed with Tsang/Dames subcohorts
Flynn work-related stress KAP cohort	Flynn et al. (22) paper 1; Flynn et al. (23) paper 2	Ketamine	Frontline healthcare workers/first responders with stress, burnout, PTSD/depression/anxiety symptoms	16 and/or 32 depending paper	32	Related Flynn reports mapped together if same cohort/protocol
Swiss psycholytic therapy follow-up	Gasser (25)	MDMA and LSD	Broad psychiatric/psychotherapeutic indications	121	121	Distinct clinical-practice follow-up
Yaqui ayahuasca community mental health program	Giovannetti et al. (35)	Ayahuasca	Depression, anxiety, grief, PTSD, substance use disorder	37	37	Distinct observational cohort
Hausner and Doležal LSD psychotherapy cohort	Hausner and Doležal (36)	LSD	Mixed psychiatric/neurotic indications	18 LSD-treated	18	Distinct historical cohort; controls not included in treated-count denominator unless specified
Jensen alcoholism treatment program	Jensen (26)	LSD	Alcohol use disorder	125	125	Distinct historical cohort
HOPE cancer psilocybin group therapy trial	Lewis et al. (14) outcome paper; Lewis et al. (16) group-format observations paper	Psilocybin	Cancer-related depressive disorder	12; qualitative subset/process report	12	Outcome paper treated as parent cohort; qualitative/process paper not double-counted
Frontline healthcare provider MBSR + psilocybin RCT	Lewis et al. (32)	Psilocybin	Frontline healthcare providers with COVID-related depression/burnout	25 randomized; 13 psilocybin arm	13	Count depends on denominator; treated-only count uses 13
Swiss MDMA/LSD group therapy model	Oehen and Gasser (28)	MDMA and LSD	Complex PTSD/trauma-related disorders	50	50	Distinct clinical-practice cohort/model
Eating disorder residential G-KAP case series	Robison et al. (10)	Ketamine	Eating disorders with comorbid depression/anxiety	5	5	Distinct case series
Frontline healthcare worker KAP case series	Robison et al. (39)	Ketamine	COVID-related burnout/PTSD	10	10	Distinct case series
Takiwasi ATOP cohort	Rush et al. (38)	Ayahuasca	Substance use disorder treatment program	52	52	Distinct observational cohort
Kindred KAP + CPT pilot	Sevelius et al. (41)	Ketamine	Transgender/gender-expansive adults with identity-based trauma	8	8	Distinct mixed-methods pilot
Smart controlled LSD alcoholism study	Smart et al. (34)	LSD	Alcohol use disorder	10 LSD-treated; 30 total	10	Treated-only count uses LSD arm
Spencer permissive LSD group therapy	Spencer (29)	LSD	Broad psychiatric/neurotic indications	10	10	Distinct historical case series
Sokolik LSD group therapy cohort	Sokolik and Hausner (30)	LSD	Compulsive neuroses/borderline cases	18	18	Distinct historical cohort
Thomas First Nations ayahuasca retreat study	Thomas et al. (40)	Ayahuasca	Problematic substance use and stress	12 analyzed	12	Distinct observational cohort
Klinik Südhang psilocybin-assisted group psychotherapy program	Wopfner and Soravia (27)	Psilocybin	Treatment-refractory substance use disorders	36 patients/89 treatments	36	Count patients once, not treatment cycles

Categorized by study method, there were seven descriptive papers (24–30) 12 quantitative papers (15–17, 20, 22, 23, 31–36), four qualitative papers (14, 18, 21, 37), and seven mixed methods papers identified (10, 11, 19, 38–41). By study type, there was only one randomized control trial (32), eight papers offering follow-ups or substudies to a trial (14, 16, 18, 21, 31, 32, 38, 39). There were four observational prospective cohort papers (16, 19, 20, 40) and four retrospective papers (15, 17, 22, 23). There were 13 case studies or series (24–30, 33–37, 41).

Demographic reporting was inconsistent across the included papers. Race and ethnicity were reported in a minority of studies; among those that did report these data, participants were most often White. After de-duplicating obvious overlapping cohorts, the age calculation includes 398 unique/less-overlapping participants, with a weighted average age of 43.69 years. Across the 30 papers, 18 papers reported gender ratios, and eight papers reported race and ethnicity ratios. There were 313 females and 184 males reported. Several papers represented overlapping cohorts so gender counts should be interpreted as paper-level reporting rather than unique participant totals.

Indications for psychedelic assisted therapies (PaT) were varied. Some papers state clear DSM-V diagnoses while others included in this review published in the late 1900s listed more vague psychiatric indications. Cancer-related depression/distress appears in five papers (16, 20, 21, 31). Healthcare-worker/first-responder distress, PTSD, burnout, depression, or anxiety in eight papers (11, 16–20, 22, 39); substance use/addiction/alcoholism in eight papers (24–27, 33–35, 40); trauma-focused indications in three papers (28, 37, 41); eating disorders with mood/anxiety comorbidity in one paper (10); broad psychotherapeutic/neurotic/psychiatric indications in four papers (15, 29, 30, 36).

### Evolution of psychedelic assisted therapy

Studies published before the 2000s primarily investigated MDMA and LSD as molecules of interest while recently there has been much more interest in ketamine and psilocybin protocols. Most programs run before the 2000s ranged in length from 1 to 18 months contrasted with programs after the 2000s which were between 1 and 12 weeks in length. Very few programs undertaken before the 2000s reported any preparation therapy for participants. The preference instead was on an experimental design with observational details documented and reviewed during the equivalent of integration therapy post-medicine (29). The patient-physician relationship was also seen as dynamic in some studies where the therapist would participate as part of the group, live on the ward, and prompt transference from patients (30). Most earlier researchers focused on personality disorders and alcohol use disorder as diagnoses for inclusion into their respective studies compared to the more recent emphasis on treatment of end-of-life distress and mood disorders (11, 20). Therapeutic interpretation was also more in keeping with traditional psychoanalytic therapy in earlier papers regardless of the indication for treatment (24, 29, 30). Individual therapy in addition to group therapy was rarely part of protocol in earlier papers and was done only at the request of patients whereas more recent publications regularly report the integrated use of individual and group therapy modalities.

### Preparation and integration

The number of preparatory therapy sessions ranged from one to five (Table 2). Generally LSD and MDMA protocols cited one to two sessions, psilocybin cited two to three sessions, and ketamine one to five sessions, sometimes broken up into individual and group when more than one was offered. Ratio of therapist to participant ranged in some studies from one to one and in others one therapist to nine participants. Most protocols divided patients into groups ranging from three to twelve patients each during preparation and integration sessions. The number of integration sessions ranged from one to eight with most studies reporting two to three sessions and no difference between psychedelic molecules of interest. Both preparation and integration therapies ranged from 1 to 2 h in length with integration therapy starting as early as 6 h after medicine and most beginning the day after.

### Medicine session

Eight studies were published on psilocybin-assisted therapy (14, 16, 20, 21, 27, 31, 32, 37), 10 on ketamine (10, 11, 15, 17–19, 22, 39, 41), and seven on LSD (24, 25, 28, 30, 33, 34, 36), and three on ayahuasca (35, 38, 40). Two of the studies that reported using LSD also used MDMA as a molecule of interest (25, 28) (Table 3). While psilocybin, MDMA, and LSD was consumed orally (PO), ketamine was provided either intramuscularly (IM) or sublingually (SL). Interestingly, no group therapy protocols used IV ketamine. Papers completed with patients with a cancer diagnosis offered a 25 mg dose of psilocybin in oral formulation (20, 21).

**Table 3:** Intervention characteristics table.

Study ID	Participant: therapist ratio	Preparation session number and length	Medicine type	Medicine format (IM/PO/SC/IV)	Medicine dosage	Integration session number and length	Individual therapy
Agin-Liebes et al. (37)	Individual psilocybin session: 1:2; group sessions: group + 2 clinician facilitators	Four group therapy sessions before psilocybin; length not specified in Agin-Liebes paper	Psilocybin	PO	0.3–0.36 mg/kg	1; 2-h individual psychotherapy/integration session the day after psilocybin, plus 4–6 additional group therapy sessions; group-session length not specified	Yes—one 8-h individual psilocybin session with two clinician facilitators, followed by a 2-h individual psychotherapy/integration session; also included group preparation and group integration
Agrawal (2023), Agrawal et al. (31)	3:3 or 4:4 participant:assigned therapist; plus 2 lead clinicians monitoring	Two prep visits: 1 × 2-h individual prep; 1 × 2-h prep day before dosing consisting of 75-min group + 45-min individual	Psilocybin	PO	25 mg	Two integration visits: each 2 h, consisting of 75-min group + 45-min individual; day 1 and week 1 post-treatment	Yes—single dose psilocybin 25-mg PO with 2 preparation and 2 integration sessions
Beaussant et al. (21)	3:5 or 4:6 overall during group components; 1:1 therapist-to-participant for simultaneous individual sessions	Two preparation visits: 1 × 2-h simultaneous individual session; 1 × 2-h prep visit consisting of 75-min group + 45-min simultaneous individual	Psilocybin	PO	25 mg	Two integration visits: each 2 h, consisting of 75-min group + 45-min simultaneous individual; day 1 and week 1 post-dosing	Yes—individual sessions occurred at visits 1–5, including individual preparation, dosing, and integration; dosing was a 6–7 h simultaneous individual session with 1:1 therapist support
Cheek et al. (24)	8:1–2 (but only 2/8 participants on LSD at a time)	1; 2 h	LSD-25	PO (taken orally in distilled water)	100 mcg, 100 mcg−300 mcg (sessions 1 and 2, respectively)	8; every Tuesday and Thursday of the 4 week program after two men were given LSD-25	Yes—individual therapy part of the standard program
Chwelos et al. (33)	1:1	Not reported	LSD	PO	200–400 mcg	Not reported	No
Dames et al. (11)	6–9:2	5; 4 2-h virtual CoP group sessions for 4 weeks and 1 individual session	Ketamine	IM	1–1.5 mg/kg	1–3; 2 h	No
Flynn et al. (22, 23)	(i, ii) Six participants: three facilitators	One group preparation/intro session in week 1; length not specified	Ketamine	SL and IM	Not fixed/reported; first dose determined by PMHNP and adjusted based on feedback/observed effect	Three group integration sessions in weeks 3, 5, and 7; length not specified	No—primarily group KAP; supportive individual coaching was provided after group sessions, but the intervention was not structured as individual therapy
Gasser (25)	NS	NS	MDMA/MDMA and LSD	NS	MDMA: 125 mg/MDMA and LSD: 125 mcg MDA for first 3 or 4 sessions then 100–400 mcg LSD dosages	NS	None
Giovannetti et al. (35)	Up to 20 participants per ceremony; therapist/facilitator ratio not specified	Preparation support was provided before each ayahuasca ceremony; exact number/length not specified	Ayahuasca	PO	Not specified	Integration support was provided after each ayahuasca ceremony; exact number/length not specified	Yes—participants received weekly individual psychotherapy, plus group therapy once or twice per month and ayahuasca ceremonies
Hausner and Doležal (36)	Group session: 12 active test-group patients in a larger 30-person therapeutic-community group, with 1 group leader/therapist + 8 observers; individual sessions appear 1:1 therapist-patient, though exact ratio not explicitly stated	Preparatory stage described, but number/length not specified	LSD	SC	100 γ/μg for individual interviews; 50 γ/μg for group sessions	Post-intoxication interpretation could occur during the session or the next day using tape/written recollections; exact number/length not specified	Yes—individual LSD psychotherapy interviews were conducted; best results were reported for individual LSD interviews combined with stay in the therapeutic community
Lewis et al. (14, 16)	(i, ii) 4–6:6	3; 120 min	Psilocybin	PO	25 mg	3; 120 min (90 min group, 30 min individual)	Yes−30 min designated for 1-on-1 breakout sessions with assigned individual therapists
Lewis et al. (32)	Approx. 3–5 participants: 3–5 assigned therapists; each participant was paired with an individual therapist	Three group preparatory sessions over 1 week; exact length not specified; each included a 30-min 1:1 breakout with assigned therapist	Psilocybin	PO	25 mg	Three group integration sessions over 2 weeks, on days 2, 6, and 13 post-dosing; exact length not specified; each included a 30-min 1:1 breakout	Yes—group PAP plus assigned individual therapist; preparatory and integration sessions included 30-min one-on-one breakouts
Oehen and Gasser (28)	12:3	1–2; one 3-h group session and at least one individual session if needed	MDMA/LSD	PO OR (MDMA)/PO (LSD)	MDMA: 25 mg or 100 mg/LSD: 25 mcg or 100 mcg	3; 6–7 h group debrief after medicine (day 2), 4 h group integration session (day 3) and at least one individual session	Yes—Those who combined group and individual setting (nine of 50). Only six out of 50 patients were treated solely in an individual setting. Some reasons for adding individual sessions were momentary excessive demands in an intense therapeutic phase, or the wish to explore the issue of fear of closeness to others
Jensen (26)	1:1	NS	LSD-25	NS	200 gamma	NS	Yes—“minimum of individual psychotherapy” details not reported
Robison et al. (39)	NS	1	Ketamine	SL (session 1 and 2)/IM (session 3)	125–200 mg, 250–275 mg, 70–100 mg (sessions 1, 2, and 3, respectively)	2; 1 week after initial dosing and 1 week after last dosing	No
Robison et al. (10)	4:3	NS	Ketamine	IM	25 mg, 25–40 mg, 40–60 mg, 40–100 mg, (sessions 1, 2, 3, and 4, respectively)	“Brief processing after group medicine session”	Yes—individual psychotherapy sessions, two times/week
Rush et al. (38)	Not specified	Preparation was part of the broader residential treatment program; exact number/length not specified	Ayahuasca	PO	Not specified	Integration/therapeutic sessions occurred during the program; exact number/length not specified. Participants had a median 31 therapeutic sessions overall	Yes—program included individual and group psychotherapy within a therapeutic community
Sevelius et al. (41)	Eight participants: three therapists	One group preparation session; 2 h	Ketamine	SL	Starting dose 5 mg/kg ketamine sublingual rapid-dissolving tablets; 100 mg SL boosters available; dose adjusted as needed	Four group integration sessions; 1.5 h each	No formal individual psychotherapy—all therapy sessions were group-based, but participants had an abridged individual intake and four 30-min 1:1 coaching calls for CPT homework support
Smart et al. (34)	1:2	NS	LSD/ephedrine sulfate	NS	LSD: 800-μg Ephedrine sulfate: 60-mg	NS	“Opportunities for individual casework and psychiatric interviews [available]”
Spencer (29)	10:2	NS	LSD	NS	200–1,500 y	NS	Yes—private interviews 1 × a week at request of patients
Sokolik and Hausner (30)	18:2	NS	LSD	NS	100–800 y	NS	“Patients with compulsive neuroses and borderline cases had individual extra seances three to four times weekly”
Thomas et al. (40)	Group retreat; 12 participants in first retreat and 6 new participants in second retreat; ceremonies led by 1 Shipibo master ayahuasquero + 3 apprentice ayahuasqueros; retreat team also led group therapy	Group orientation several hours before retreat + group counseling/reflection during Day 1 and Day 2 before first ceremony; exact session lengths not specified	Ayahuasca	PO	Small glass of ayahuasca, approximately 50–100 mL per ceremony; alkaloid dose not chemically quantified	Group debrief/integration the morning after each ceremony, plus final debrief on Day 4; exact session lengths not specified.	No formal individual therapy—intervention was group counseling/retreat plus individual ceremonial interactions with ayahuasquero, but not structured individual psychotherapy
Tsang et al. (17) (i) “A Pilot Study…” (ii) “Safety and tolerability…” Tsang et al. (19); Tsang et al. (18)	6–9:2	(i) Weekly 2-h sessions (ii) 1–3 in addition to weekly group sessions	Ketamine	SL/IM	(i) 100–300 mg (SL) for first session; 1.0–1.5 mg/kg (IM) for next 2 sessions (ii) 100–300 mg (SL) for first session of cohorts 1, 2 and 3; 0.5–1.6 mg/kg (IM) for the rest of the sessions	(i) Weekly 2-h sessions (ii) 2–3 along with online RTT sessions	No
Wopfner and Soravia (27)	Mean group size 6.75 participants; group sessions led by 2 psychotherapists + 2 co-therapists/team members	Five prep sessions total: 3 individual + 2 group; session length not specified	Psilocybin	PO	First participation: max 25 mg; repeat participation: up to 40 mg	Four integration sessions total: three group sessions at day 1, week 1, and week 2 post-psilocybin + 1 individual session in the week after dosing; session length not specified	Yes—the program includes individual preparation sessions and one individual integration session, plus group preparation, group psilocybin, and group integration

IM, intramuscular; PO, oral; SC, subcutaneous; IV, intravenous; NS, not specified.

### Stratified summary by substance class, diagnosis, and group format

LSD and MDMA/LSD studies were largely historical or clinical-practice reports. LSD studies most often involved alcohol use disorder, compulsive neurosis, broad psychiatric indications, or mixed inpatient psychotherapeutic populations. These studies varied substantially in dose, setting, duration, and degree of individual psychotherapy, and many lacked standardized outcome measures. The Swiss MDMA/LSD clinical-practice model differed from earlier LSD studies by focusing largely on complex PTSD and trauma-related disorders, using MDMA and LSD in a structured group-psychotherapy model with individualized clinical judgment. Ayahuasca studies were primarily observational or naturalistic and were embedded in ceremonial, community, residential, or culturally specific treatment settings, most often for substance use disorders and community mental health symptoms. These studies reported improvements in depression, anxiety, trauma symptoms, substance-use outcomes, quality of life, and connectedness, but interpretation is limited by uncontrolled designs and difficulty separating the effects of ayahuasca from the broader therapeutic, cultural, ceremonial, and residential context.

Among the included studies, a smaller number of studies utilized KAT, which was mostly derived from a small number of related cohorts, while the majority involved classic serotonergic psychedelics (e.g., psilocybin, LSD, MDMA). Outcomes were broadly consistent across substance classes in demonstrating improvements across psychiatric symptoms and functioning. However, ketamine-based studies more frequently emphasized feasibility within structured group formats (10, 11, 15, 17–19, 22, 39, 41), whereas studies involving serotonergic psychedelics more often reported sustained improvements and longer-term follow-up data, including changes in psychological wellbeing and social connectedness (24, 25, 28, 30, 33–36, 38, 40). Compared with classic psychedelic studies, ketamine papers more frequently emphasized pragmatic implementation, safety, tolerability, and feasibility of multi-session group-based care.

### MMAT scores

Using the MMAT 2018 framework, methodological quality across the 30 papers was heterogeneous (Supplementary Appendix S2). The papers scoring highest were recent studies with prospective designs, structured methods, and clearly described study designs (15, 16, 18, 21, 32, 37, 38, 41). In particular, the Beaussant qualitative acceptability study and Sevelius mixed-methods pilot were among the highest-quality papers. These authors clearly aligned aims, data collection, analysis, interpretation, and methodological transparency (21, 41). The Lewis et al. (32) randomized controlled trial was the strongest quantitative efficacy design, although it was limited by small sample size, limited blinding, and unequal intervention intensity. Several recent open-label or observational studies, including Agrawal, Lewis HOPE outcomes, Rush/ATOP, Tsang safety review, and Tsang firefighter studies, were appraised as moderate because they used validated outcomes and clear procedures but lacked randomization, control groups, or robust confounder control (10, 15, 16, 18–20, 31, 38). Most historical LSD-era papers were appraised as low quality by contemporary MMAT standards because of unclear sampling, limited standardized outcome measurement, incomplete reporting, and uncontrolled or poorly controlled designs (24, 25, 28, 30, 33, 34, 36). Overall, the evidence base is best characterized as promising but methodologically early, with a small number of higher-quality modern studies and a larger body of descriptive, uncontrolled, or historical reports that are valuable for context but limited for causal inference. The quality of the quantitative papers was negatively affected by unrepresentative samples that were often volunteer based. Amongst the mixed methods studies there was a common theme of inadequate integration and discussion of qualitative and quantitative components. While the papers presented strengths according to criteria from the MMAT the quality of the papers were compromised due to factors such as unrepresentative samples, unstandardized data collection methods and inadequate interpretation of results.

### Clinical improvements

Across the 30 papers, 15 papers reported pre–post quantitative outcomes using validated mental health, functioning, substance-use, or wellbeing measures, representing approximately 13 unique study cohorts after accounting for clearly overlapping papers (Table 4). The most frequently used measures were the Generalized Anxiety Disorder-7 scale (GAD-7) in seven papers, Patient Health Questionnaire-9 (PHQ-9) in six papers, and the Posttraumatic Stress Disorder Checklist for DSM-5 (PCL-5), reported in eight papers. Other validated outcomes included the Montgomery–Åsberg Depression Rating Scale (MADRS) and Hamilton Anxiety Rating Scale (HAM-A) in two cancer papers, the Hamilton Depression Rating Scale (HAM-D) in one HOPE cancer trial paper, the Beck Depression Inventory/Beck Depression Inventory-II (BDI/BDI-II) and Beck Anxiety Inventory (BAI) in two ayahuasca studies, the Brief Inventory of Psychosocial Functioning (B-IPF) in two ketamine studies, and additional measures such as the Quick Inventory of Depressive Symptomatology–Self-Report, 16-item version (QIDS-SR-16), State-Trait Anxiety Inventory (STAI), Maslach Burnout Inventory (MBI), Demoralization Scale-II (DS-II), Watts Connectedness Scale (WCS), Addiction Severity Index (ASI), World Health Organization Quality of Life-BREF (WHOQOL-BREF), World Health Organization Quality of Life Spiritual, Religious and Personal Beliefs scale (WHOQOL-SRPB), Inventory of Complicated Grief (ICG), Mystical Experience Questionnaire-30 (MEQ-30), and Emotional Breakthrough Inventory (EBI) in one or more papers. Overall, 14 of the 15 papers using validated pre–post measures reported some degree of clinical improvement in depression, anxiety, posttraumatic stress disorder symptoms, functioning, substance-use severity, quality of life, burnout, demoralization, connectedness, or related outcomes. Several studies reported statistically significant improvements, including the cancer psilocybin trials using MADRS, HAM-A, or HAM-D outcomes (20, 31); ketamine-assisted group therapy studies using PHQ-9, GAD-7, PCL-5, and B-IPF outcomes (17, 19); the ayahuasca observational studies using BDI/BAI or addiction-related measures; and the randomized trial of psilocybin-assisted psychotherapy plus mindfulness-based stress reduction, which found greater reduction in depressive symptoms compared with mindfulness-based stress reduction alone (32, 35, 38). In contrast, Smart et al.'s (34) study did not identify clear clinical or statistical benefit. Several additional papers reported clinical improvement based on qualitative findings, participant feedback, or clinician judgment, but did not use standardized pre–post statistical testing and therefore were not counted among the validated quantitative outcome papers.

**Table 4:** Validated screening tools for quantitative outcomes.

Study ID	GAD-7 outcomes	PHQ outcomes	MADRS	HAM-D/QIDS	VAS	PCL-5/trauma scale	Other validated scale outcomes
Agrawal, 2023/Agrawal et al. (20)	N/A	QIDS-SR used; decreased 5.9 points, approximately 49% reduction	Decreased 19.2 at week 3; decreased 19.1 at week 8; 80% sustained response; 50% remission	N/A	Depression VAS decreased 46.2 points; 53% decrease in self-rated depression severity	N/A	HAM-A decreased approximately 17 points; STAI-S and STAI-T each decreased 17.2 points
Agrawal et al. (20, 31) long-term follow-up	N/A	N/A	At 24 months: average decrease of 15.0 from baseline; 53.6% significant depression reduction; 50% sustained depression reduction/remission	N/A	N/A	N/A	HAM-A decreased 13.9 from baseline; 46.4% significant anxiety reduction; 42.9% sustained anxiety reduction
Dames et al. (11)	Pre aggregate: 12; post: 6; 91% improved among those screening positive	Pre aggregate: 13; post: 7; 79% improved	N/A	N/A	N/A	Pre aggregate: 47; post: 20; 86% of those screening positive for PTSD screened negative after treatment	B-IPF pre: 42; post: 18; 92% reported clinically significant life/work functioning improvement
Flynn et al. (22)	Median 9.5 to 6; *p* = 0.003	Median 12 to 5; *p* < 0.001	N/A	N/A	N/A	Median 27 to 10; *p* < 0.001	Maslach Burnout Inventory subcomponents improved; MEQ-30 increased across sessions
Lewis et al. (14) HOPE clinical outcomes	N/A	N/A	N/A	HAM-D baseline 21.5 to 10.09 at 2 weeks, *p* < 0.001; baseline 21.5 to 14.83 at 26 weeks, *p* = 0.006	N/A	N/A	Six of 12 met remission criteria at 2 weeks; eight of 12 showed clinically substantial change
Lewis et al. (32) RCT	N/A	N/A	N/A	QIDS-SR-16: MBSR + psilocybin improved more than MBSR alone at 2 weeks; between-group effect 4.6, 95% CI 1.51–7.70, *p* = 0.008; effect waned by 6 months	N/A	PCL-5 used as secondary outcome; favored psilocybin arm but was not the primary endpoint	MBI-HSS-MP, DS-II, and Watts Connectedness Scale used; secondary outcomes generally favored psilocybin arm but many did not survive multiple-comparison correction
Robison et al. (10)	Two of five had clinically significant GAD-7 improvement; examples: 18 to 8 and 12 to 0	Four of five had clinically significant PHQ-9 improvement; examples: 20 to 0 and 25 to 2	N/A	N/A	N/A	N/A	No serious adverse events; depression/anxiety changes varied by participant
Robison, 2024/Robison et al. (39)	Mean 11 to 7; 36% reduction; 8 of 10 improved	Mean 12 to 5; 58% reduction; 90% minimal/mild or clinically improved	N/A	N/A	N/A	Mean 34 to 14; 59% reduction; 100% screened negative post-treatment	MEQ-30 and EBI collected during ketamine sessions; MEQ increased in third IM session
Tsang et al. (15), A Pilot Study	+KaT: decreased 5.90; -KaT: decreased 4.90; total: decreased 5.56	+KaT: decreased 7.08; -KaT: decreased 6.21; total: decreased 6.79	N/A	N/A	N/A	+KaT: decreased 15.97; -KaT: decreased 12.53; total: decreased 14.83	No significant difference between +KaT and -KaT after adjustment; improvements occurred in both groups
Tsang et al. (15), Safety and tolerability	No validated symptom outcome scores reported	No validated symptom outcome scores reported	N/A	N/A	N/A	No validated symptom outcome scores reported	Safety/tolerability outcomes only; adverse events and physiological measures reported
Tsang et al. (19), firefighter prospective case series	Significant reduction post-treatment; persisted at 6 months, but did not survive Bonferroni correction	PHQ-9 not significantly different pre-to-post or at 6 months	N/A	N/A	N/A	Significant PCL-5 reduction post-treatment; persisted at 6 months, but did not survive Bonferroni correction	B-IPF improved post-treatment and at 6 months; did not survive Bonferroni correction
Giovannetti et al. (35)	N/A	BDI-II 15.7 to 5.1 after two ceremonies	N/A	N/A	N/A	PCL-5 used; paper reports marked PTSD symptom reductions, exact change not in abstract	BAI 16.6 to 6.3; ICG 39.6 to 10.7; seven of eight suicide-risk cases resolved after one ceremony
Rush et al. (38)/ATOP Takiwasi	N/A	BDI used; depression improved significantly from baseline to 1-year follow-up	N/A	N/A	N/A	N/A	ASI, BAI, WHOQOL-BREF, WHOQOL-SRPB used; significant improvements in alcohol/drug use severity, anxiety, depression, and some quality-of-life domains
Sevelius et al. (41)	GAD-7 14.63 to 8.75; *p* = 0.030; Cohen's *d* = 0.96	PHQ-8 16.13 to 9.75; *p* = 0.019; Cohen's *d* = 1.08	N/A	N/A	N/A	Adapted PCL-5 used; trauma-symptom changes were in expected direction but not statistically significant	Cognitive fusion 6.20 to 4.66; *p* = 0.002; Cohen's *d* = 1.78
Thomas et al. (40)	N/A	N/A	N/A	N/A	N/A	N/A	Significant improvements in mindfulness, empowerment, hopefulness, quality-of-life meaning, and quality-of-life outlook; significant reduction in cocaine use

### Depression

Across the 30 papers, depression was most commonly assessed using the PHQ-9, MADRS, HAM-D, QIDS-SR/QIDS-SR-16, BDI/BDI-II, and PHQ-8 in 13 papers. Most papers that measured depression quantitatively reported improvement after treatment, although the strength of evidence varied substantially by design. The largest and most consistently quantified improvements were reported in the psilocybin cancer studies and ketamine-assisted group therapy studies (14, 16, 18–20, 31). In the Sunstone cancer trial, MADRS scores decreased by approximately 19 points by week 8, with 80% sustained response and 50% remission; the 24-month follow-up found that 53.6% continued to show significant depression reduction and 50% had sustained reduction/remission (20, 31). The HOPE cancer trial similarly reported HAM-D reductions from 21.50 to 10.79 at 2 weeks, with continued improvement at 26 weeks (9, 14). Among ketamine studies, PHQ-9 improvements were reported in healthcare-provider, frontline-worker, eating-disorder, and community-of-practice cohorts, including reductions in both Flynn and Robison's frontline healthcare-worker case series, and a 6.79-point PHQ-9 decrease in Tsang's CoP/KAT pilot (10, 11, 17–19, 22, 23, 39). Ayahuasca studies also reported depression improvements, including a BDI-II decrease from 15.7 to 5.1 in the Yaqui community program and significant BDI improvement at 1-year follow-up in the Takiwasi ATOP cohort (35, 38, 40). Overall, depression outcomes were broadly positive, but many studies were open-label, uncontrolled, retrospective, small, or overlapping in sample, so the depression findings should be interpreted as promising rather than definitive.

Dames et al. (11) saw a reduction of PHQ9 scores from 13 to 7 (*n* = 62); they reported that 79% saw improvements in depression that lowered participants into a milder category or had significant clinical improvement. Tsang et al. (17) reported a decrease of 7.08 points on the PHQ9 for the ketamine group compared to a decrease of 6.21 points for the non-ketamine group. They found clinically and statistically significant reductions in PHQ9 with large effect sizes among participants in both arms, but did not detect statistically significant differences in changed-scale scores between the arms (31). Robison et al. (39) noted a 58% reduction in average PHQ-9 scores: 90% had minimal/mild depression or clinically significant improvement. In his 2022 paper on patients with eating disorders, Robison et al. (10) shares that four of the five participants experienced clinically significant improvements on the PHQ-9 score.

Lewis' HOPE trial noted clinical and statistically significant decreases in HAM-D scores from baseline to 2- and 26-week time point; six out of the 12 participants met criteria for remission at 2 weeks as defined by HAM-D < 7, three out of 12 demonstrated a clinically significant change (4–6 points), and eight out of 12 demonstrated clinically substantial change (7–12 points) (9). They also reported that participants who achieved a complete mystical experience as per MEQ-30 during the psilocybin session, had significantly greater decreases in HAM-D (9). Agrawal et al. (20) noted a 80% sustained response to psilocybin treatment with 50% at full remission from depressive symptoms at week 1 which was subsequently sustained for 8 weeks. This was found to be both clinically and statistically significant. He also reported a reduction in MADRS scores from baseline to post treatment of 19.1 points by week 8. The Quick Inventory of Depressive Symptomatology—Self-Report scores showed a mean decrease of 5.9 which was clinically significant, a 49% reduction in self-reported depressive symptoms from baseline to week 8. Maudsley VAS detected a change of −46.2, a 53% decrease in self rated depression severity; mean reduction of 17.0 on the HAM-A; VAS showed mean change of −1.2 (9).

### PTSD

Across the 30 papers, eight reported use of a PTSD or trauma-related symptom scale. They were most commonly measured with the Posttraumatic Stress Disorder Checklist for DSM-5 (PCL-5), especially in the KAT studies. Overall, studies that quantitatively assessed PTSD symptoms generally reported improvement. In the Dames 2022 study, PCL5 scores decreased from 46 to 20 (*n* = 55); 86% of those who screened positive for PTSD now screen negative (11). This was clinically significant. Flynn's et al. (22) group KAT study reported a significant median PCL-5 reduction (*p* < 0.001) among healthcare workers and first responders. Tsang et al. (17) reports a decrease of 15.97 points on the PCL5 for the ketamine group compared to 12.53 points for the non-ketamine group, but was not statistically significant in the changed-scale score between the two arms. Robison et al. (39) used a PCL-5 score of 31 to determine if a participant met criteria for PTSD and noted a 59% reduction in average PCL-5 scores. At the end of treatment in the study, 100% screened negative for PTSD. Although these papers did not report the use of a scale, outside of ketamine studies, Giovannetti's Yaqui ayahuasca-assisted program reported reductions in PTSD symptoms, and several qualitative or clinical-practice papers described trauma-related improvement, including Agin-Liebes' psilocybin-assisted group therapy study in gay men with HIV-related trauma, Oehen's Swiss MDMA/LSD group model for complex PTSD and trauma-related disorders, and Sevelius' Kindred KAP+CPT pilot for identity-based trauma (28, 35, 37, 41).

### Anxiety

Eleven papers reported the use of a validated anxiety scale with the GAD-7 used in seven papers (10, 11, 15, 17, 19, 22, 39, 41), HAM-A used in one paper (31), the BAI in two (35, 38), and both HAM-A and STAI in one paper (20). The largest quantified improvements were reported in the psilocybin cancer studies, where HAM-A scores decreased by approximately 17 points at 8 weeks and remained improved at 24 months (20). Ketamine-assisted group therapy studies also consistently reported anxiety reductions, including Dames' et al. (11) finding that 91% of participants with baseline generalized anxiety symptoms improved, Flynn's median GAD-7 decreased from 9.5 to 6 and 14 to 7 in their two papers (22, 23). Robison's et al. (10) frontline healthcare-worker case series showed a 36% GAD-7 reduction with 60% reporting only minimal/mild anxiety or clinically significant improvement at post-treatment in their 2023 paper. In their 2022 paper, they noticed two of five participants experienced clinically significant improvements in GAD-7 score with significance recorded as a change by 4 points (10). Sevelius' et al. (41) Kindred pilot showed GAD-7 improvement from 14.63 to 8.75, and Tsang's CoP/KAT pilot showed a 5.57-point GAD-7 decrease. Ayahuasca studies also reported anxiety improvements, including Giovannetti's et al. (35) BAI decrease from 16.6 to 6.3 and Rush's significant anxiety improvement at 1-year follow-up. Overall, anxiety outcomes were consistently positive among papers that measured them, but most studies were small, uncontrolled, open-label, retrospective, or observational, so the findings should be interpreted as promising but preliminary.

### Overall psychological wellbeing

Several studies used broader functioning, quality-of-life, burnout, demoralization, connectedness, disability, or participant-rated improvement measures, including the BIPF, Sheehan Disability Scale, EQ-5D-5L, Demoralization Scale-II, Maslach Burnout Inventory, Watts Connectedness Scale, World Health Organization Quality of Life-BREF, and World Health Organization Quality of Life Spiritual, Religious and Personal Beliefs scale. Overall, findings were generally favorable.

Reported results for general psychological wellness, though positive, were incredibly heterogeneous with no two studies utilizing the same scale or approach. Dames reported a decrease in BIPF scores from 42 to 18 (*n* = 65) with 92% of participants reporting clinically significant life/work functionality improvements (11). Similarly, Spencer (29) noted three improved sufficiently and required no further psychiatric help; three unimproved; four helped to a fairly definite degree; one patient on 2/week individual LSD and improving. Sokolik and Hausner (30) noted 12/18 completed the full course; six quit; two had no results (borderline); four fair improvement; six substantial improvements in symptoms and interpersonal relations.

Agrawal et al. (20) noted that the post intervention EuroQol-5-dimension 5-level scale showed mean difference of −2.3; Demoralization Scale II showed a decline of −6.8 points; Sheehan Disability Scale showed mean difference of −7.5; STAI-S and STAI-T showed a mean difference of −17.2. Gasser (25) shares good improvement in 65% of participants. They claim that PaT had the biggest impact on participants' emotional lives (65.4%); interpersonal lives (56.2%); biological insights (48.8%); and life decisions (36.4%) (38). There was a notable decrease in substance use, improvement in quality of life, spiritual life, and social behavior. Lewis et al. (32) reported that burnout, demoralization, and connectedness outcomes favored psilocybin-assisted psychotherapy plus mindfulness-based stress reduction compared with mindfulness-based stress reduction alone, although these secondary outcomes did not survive correction for multiple comparisons. Rush's et al. (38) Takiwasi ayahuasca cohort reported significant improvements in substance-use severity, depression, anxiety, and some quality-of-life domains at 1-year follow-up, while Thomas et al. (40) reported improvements in hopefulness, empowerment, mindfulness, quality-of-life meaning, and substance-use outcomes in a First Nations ayahuasca-assisted therapy cohort. Historical and descriptive studies also reported global clinical improvement, such as Gasser's (25) report of improvement in emotional life, interpersonal life, biological insight, and life decisions, Spencer's (29) clinical observations of psychiatric improvement in some participants, and Sokolik and Hausner's (30) report of fair to substantial improvement in symptoms and interpersonal relations. However, these older reports relied largely on clinical judgment or participant self-report rather than standardized psychometric testing. Overall, broader psychological wellbeing outcomes were consistently positive but difficult to synthesize quantitatively because measures, timepoints, populations, and analytic methods varied substantially across studies.

### Between-group differences in studies with comparison groups

Six papers used comparison groups who either received a substitute medication to simulate the effects of psychedelics or who received the group therapy intervention only (14, 17, 24, 26, 34, 36).

Lewis et al.'s (32) randomized controlled trial was the only modern RCT among the six comparison-group papers. Participants were randomized to either an 8-week Mindfulness-Based Stress Reduction (MBSR) curriculum alone or MBSR plus group psilocybin-assisted psychotherapy with 25 mg psilocybin. The primary outcome was depressive symptoms on the QIDS-SR-16 at 2 weeks post-intervention, with burnout assessed using the Maslach Burnout Inventory and secondary measures including demoralization, PTSD symptoms, and connectedness. The authors reported that MBSR plus psilocybin was associated with clinically and statistically significant reductions in depressive symptoms relative to MBSR alone at the 2-week endpoint; however, this between-group advantage waned by 6 months, when both groups reported lower depression scores. Hausner and Doležal's (36) historical LSD psychotherapy paper compared individual LSD psychotherapy, group LSD psychotherapy, placebo patients, and control patients without LSD or placebo. The results were mixed and suggested that therapeutic response depended heavily on context and format, noting that psychedelics act as non-specific amplifiers and so the effects will always be shaped by context (42, 43). Individual LSD interviews showed the greatest final symptom improvement, with symptoms decreasing to 27% of pretreatment intensity, whereas group LSD patients showed less improvement, decreasing only to 74% of pretreatment intensity.

The other papers reported improvements for participants across all treatment groups, but no clinically or statistical difference between groups. This is in keeping with the other studies in this review reporting positive correlations between improvement of psychiatric symptoms and treatment with PaT. However, there were no statistical differences between true psychedelic intervention and placebo controlled or therapy only comparison groups.

In the Smart et al. paper, there were three blinded groups—LSD, ephedrine, and therapy only (23). In Tsang et al.'s (17) pilot paper, they compared the outcomes of the group receiving ketamine to a similar group of demographic-matched individuals receiving only the group therapy intervention termed Communities of Practice. Interestingly, while both these studies did not find significant differences between the control or placebo groups, they did see a significant change in clinical outcomes from all groups. Smart et al. (34) noted that while all groups showed a significant reduction in the number of problematic drinking behaviors such as intoxication, morning drinking and impairments with work, patients in the LSD group did not present with a significant relative decrease or elimination of problematic drinking behaviors nor did it seem to increase the participant's engagement in therapy compared to the ephedrine and therapy only groups. Similarly, Tsang et al. (17) noted statistically significant reductions of the mean change in score of PHQ9, GAD7 and PCL5 for both the ketamine and group therapy only cohort with a large effect size Compared to the therapy only group, exposure to adjunctive ketamine was associated, upon adjustment, with an average change of −0.78 (95% CI: −3.28 to 1.72, *p* = 0.534) points in PHQ-9 scores; and −0.60 (CI: −2.68 to 1.49, *p* = 0.567) and −2.23 (CI: −9.79 to 5.33, *p* = 0.557) points in GAD-7 and PCL-5 scores, respectively. Linear regression models found no difference between the ketamine and group therapy only cohorts as a junction of adjunct ketamine or other covariates. Although Cheek et al.'s (24) study noted that the overall results of the LSD group were more positive compared to their therapy only intervention group in terms of problem solving, self understanding, and interpersonal relationships and decreased guilt and irritability, correlational analyses suggest that immediate attitudinal shifts were not related to long term outcomes. The authors conclude that while

“LSD proves valuable for “getting through” to the alcoholic…it is most important that early gains from LSD are maintained to 3 month mark with a recommendation for further research of re-dosage at 3 month mark.” (24)

Jensen (26) compared group-based alcoholism treatment with LSD, group therapy without LSD, and individual-treatment controls. The LSD program group had higher rates of abstinence or improvement at follow-up than the comparison groups. However, interpretation is limited by substantial selection bias, as patients in the non-LSD group included those who left early, refused LSD, or were medically unsuitable for LSD.

### Mindfulness and social wellness benefits

Qualitative, mixed-methods, or descriptive narrative data were reported in 18 papers (10, 11, 14, 18, 19, 21, 24–30, 37–41). Of these, 10 provided sufficient participant narratives, interview data, qualitative survey responses, or author-reported descriptive themes (10, 11, 14, 21, 25, 28, 37, 39–41). Four additional papers contained potentially relevant qualitative observations but lacked sufficient depth or methodological detail for robust thematic analysis (18, 19, 24, 36), while four were primarily descriptive or programmatic and did not provide enough qualitative data for thematic coding (26, 27, 29, 30). Themes included deriving personal insights about the flexibility of reality which then extended into the possibility of flexibility in personality, perception of past traumas, and disordered thinking.

The psychedelic experience increased one's sensitivity to the feelings of others, fostering empathy and decreased self-concern, allowing one to gain insight into associations and relationships that were previously difficult to process. In Agin-Liebes et al. (37), participants described shifting from “autopilot” to “meta-aware” processing and from “trauma-dominant” to “growth-dominant” narratives, with psilocybin helping them process grief, shame, and survivor guilt while accessing “joy, gratitude, love, care, compassion.” Gasser (25) reaffirmed the thought that

“the impact of their psycholytic sessions was greatest on the patients' emotional lives”.

One participant shared,

“...I have found the sessions absolutely mind shifting. The work that I have done in these sessions have made me able to process my trauma and move forward in a way I never thought would be possible…” (11).

Several studies also reported mystical, spiritual, or self-transcendent experiences as part of participants' broader wellbeing outcomes, suggesting that perceived benefits of group PaT may extend beyond diagnosis-specific symptom change to include meaning-making, spiritual connectedness, and existential wellbeing (16, 32, 38–40). Some have reported that PaT allowed them to revisit previous experiences in a confrontative, detailed, relentless manner, a “death-rebirth experience” that led to more positive self-perception and increased resiliency (32). Even if this insight was brief, participants still noted that they were helpful. One person in particular shared,

“my [eating disorder] symptoms were greatly improved during the short treatment…after that, they basically came back, but it was helpful to catch the vision of what life could be like without them.”

Hausner and Doležal (36) quoted participants who described LSD as opening “a new way” to evaluate life experiences and disrupting usual thought currents, while Cheek et al. (24) remarked that,

“LSD would appear to be a valuable tool for ‘getting through' to the alcoholic, shaking him out of his narrowly focussed pattern of egocentric, resentful, guilt-ridden, alcoholic thinking.”

Secondly, the psychedelic-assisted effect of de-centering from individual narratives to self-transcendence and increased compassion for others had lasting effects on participants, positively impacting other interpersonal relationships while enhancing personal mind-body connection. One person shared,

“I have been led through some of my fears while experiencing a newfound deep sense of love for life. I'm able to connect with my partner from a more intimate authentic place. I'm also noticing a greater depth of connection with those I work with and for.” (11)

Oehen and Gasser (28); Gasser's (25) Swiss MDMA/LSD group model also emphasized group-mediated healing in trauma-focused therapy: one participant stated that the group helped her “open up,” “become authentic,” and “build relationships,” while the authors described MDMA as increasing tolerance for trauma processing and LSD as helping address deeper relational, attachment, and self-perception patterns. This sense of social wellness was further enhanced by reports by the group therapy aspect of the respective PaT protocols. For example, a participant shares that,

“group experience helped me open up, be close to others, express myself, become authentic, and build relationships.” (9)

The opportunity to mutualize memories and healing in a group setting enriched the integration process, contributing to the validation and consolidation of insights gained from these sessions in the long term. Another participant shares that,

“I feel as though my views and feelings toward others and how they treat me/have treated me has come to a sense of understanding that I didn't feel this way previously.” (32)

In Beaussant et al.'s (21) qualitative analysis of psilocybin-assisted therapy for patients with cancer and major depressive disorder, participants described the group and simultaneous-session model as increasing safety, connection, belonging, self-transcendence, and compassion; one participant noted that seeing others' transformative processes “elicits compassion for the other individuals,” including people they might not otherwise have felt connected to. Lewis et al.'s (14) HOPE trial observations similarly described participants feeling “connected to one another on a deep” level, with one participant reporting that others' healing journeys were “woven into my own story,” and another noting that the group helped them understand they were “not alone” with cancer-related challenges. In Agin-Liebes et al. (37), participants reported forgiveness, gratitude, and belonging, including the realization that others in the group had also endured profound losses and would “come back together” after the psilocybin sessions. Across these studies, the group context appeared to support validation, normalization, peer witnessing, and integration of insights into interpersonal life; however, the strength of evidence varied, and several older or descriptive reports relied on clinical observation rather than formal qualitative methodology.

### Individual and group therapy synergies

Focusing further on the group structure, participants have consistently reported feeling a strong sense of safety, trust, and reassurance. One participant shared,

“it was good to see other people's experience and why they were there, their fears, their questions…” (21)

Although some participants reported being nervous at first, the group approach quickly fostered an atmosphere of connection and belonging. A participant in the Dames et al. study shared,

“the trust that was felt in our group occurred in the first session and has increased every week.” (11)

Participants frequently described group sessions as important in the framework of the later PaT studies, as it encouraged community-building while reserving an intentionally therapeutic space where participants could feel less defensive and fully absorbed in their own journeys. One participant noted,

“I felt like the group format psilocybin session worked well and maximized my individual therapeutic response.” (17)

Another shared that,

“the group helped to ground me.” (21)

and

“the small groups have been amazing…How could we know each other without knowing all of our stories?” (11)

Hearing from participants, it was evident that the group therapy component of the intervention was healing independent of the psychedelic component. This is further reinforced by the studies with quantitative comparisons reporting similar improvements between group therapy comparisons or control groups without adjunctive psychedelic sessions (24, 25, 34). This interpretation is further supported by the Roots to Thrive comparison study, where both the ketamine-assisted and non-ketamine Community of Practice groups improved significantly on depression, anxiety, and PTSD measures, while between-group differences favoring ketamine were larger but not statistically significant. This suggests that the group container itself may be an active therapeutic ingredient, with ketamine potentially augmenting rather than replacing the benefits of group-based relational work (17).

The newer cancer psilocybin studies suggest that the most acceptable model may not be purely individual or purely group-based, but rather a hybrid structure in which individual preparation, dosing, or integration preserves privacy and psychological containment while group preparation and integration provide shared meaning-making and belonging. Beaussant et al. (21) specifically noted that participants valued the complementarity of one-on-one sessions with group sessions, allowing therapy to remain introspective while adding a sense of “togetherness.”

While Lewis et al. (14) still claims that it is important to emphasize that variety in individual experiences can enrich rather than detract from group dynamics, there have been mixed views regarding whether the group setting could be a distraction for some people. While some have reported the ability to listen to fellow group members in psychedelic dosing sessions enhanced the sense of group connection and cohesion (14), others have pointed out that being exposed to other participants' emotions could be disruptive (21). Some participants also reported constantly comparing themselves with others, posing challenges in their treatments and self-perception. Especially notable in the studies with cancer patients was one participant who shared,

“I felt like an imposter in that group session…it's just I felt that I am on the lower spectrum for maybe the need for this.” (19)

Another remarked,

“I was the only person who had stage four cancer…I felt like I was thinking about dying more than them and I felt a little bit outside.” (21)

Nonetheless, group PaT promotes positive transference between participants and staff during the treatment process, which many have reported to be crucial to the processing of one's trauma and emotions, as well as forming healthy relationships with others (29, 39). Positive transference is defined as a patient transferring positive feelings that they previously experienced with a caregiver with the therapist or group members. The authors note,

“we have regularly observed the fact that the results of therapy were in direct proportion to the degree of emotional “getting in” of the patient into the group.” (30)

The therapeutic framework of group cohesion with personalized introspection can facilitate profound personal growth, resilience, and long-term emotional healing.

The firefighter studies add that group synergy may be especially important when participants share a professional identity or trauma culture. In Tsang et al.'s (19) firefighter cohort, participants described the benefit of processing trauma with a “knowing crowd” who understood the norms of firefighter culture, with one participant reporting that the group “expanded my ability to trust others and strangers.”

Using the segregated methodologies approach, the quantitative and qualitative findings in this review are complementary in nature though they do point toward a positive treatment outcome from group PaT. For example, in the psilocybin-assisted therapy cohort reported by Agrawal et al. (20) and Beaussant et al. (21), quantitative reductions in depressive symptoms were complemented by qualitative reports of enhanced meaning-making and emotional processing, illustrating a complementary relationship in which experiential changes provided context for observed clinical improvements. The quantitative results show an overall decrease in validated scale outcomes reflective of an improvement in depression, anxiety, trauma, and other psychiatric diagnoses. The qualitative results discuss improvements in personal mindfulness and social connectedness aided by the group format of these protocols. Both data types however point to the value of group therapy contributing toward improved personal awareness and acceptance, interpersonal relationships, as well as clinical improvement in psychiatric symptoms.

The qualitative data reliably reveal that the group aspect of PaT was helpful to participants with supportive findings from the mixed-methods integration. This, coupled with minimal differences between group therapy modalities with and without psychedelic medicines may point to the therapeutic benefit being the supportive group environment (25). Unfortunately there are few studies comparing individual therapy to group therapy in order to further test this hypothesis.

The studies included in this review mention two unique groups of participants—firstly a group where the psychedelic medicines offer some degree of “unlocking” of rigidity. Often these include participants who are resistant to therapy, or who use substances. While for another group of participants, the value of group PaT is in fact fostering the connection within the group. For these individuals, it is potentially the group therapy that allows for transformation with the psychedelic medicines allowing for an expediting of therapy through increased vulnerability and sharing which is met with connection and support through the group. Attempts were made to interrogate the quantitative data for further evidence of dramatic changes in some areas between patients but this was difficult given the heterogeneous nature of the study methodologies.

### Adverse events, sustainability, and costs

Six studies routinely collected vital signs before and/or after medicine administration (10, 15, 16, 20, 32, 39), while one additional clinical-practice paper reported monitoring vital signs only in exceptional circumstances when clinically indicated (28). Safety and tolerability reporting was more common: 12 papers explicitly reported or monitored adverse events, side effects, or serious adverse events, although the rigor of reporting varied substantially between contemporary trials and older historical reports (10, 14–16, 20, 21, 24, 28, 29, 32, 36, 39). In modern psilocybin and ketamine studies, serious adverse events were generally absent, while reported side effects were typically transient and expected. These included elevated blood pressure, nausea, vomiting, headache, dizziness, dissociation/sedation, anxiousness, and transient psychological distress. The most detailed safety data came from Tsang et al.'s (15) ketamine safety review, which reported post-treatment elevated blood pressure in 49.16% of 448 sessions, nausea in 12.05%, vomiting in 2.52%, headache in 3.35%, and dizziness in 1.56%. Contemporary psilocybin trials also monitored adverse events, suicidality, and vital signs, with Agrawal et al. reporting no psilocybin-related serious adverse events, no suicidality, and no laboratory or electrocardiogram abnormalities, and Lewis et al. reporting no serious adverse events in both the HOPE cancer study and the 2025 randomized trial (14, 20, 21, 32). Historical LSD-era papers were less standardized in safety reporting, though Cheek et al. described one severe panic reaction that was managed with reassurance, while Hausner and Spencer described acting out, aggression, and emotionally intense group processes (24, 29, 36). Across studies, barriers to implementation included treatment cost, uncertain durability of response, lack of trained practitioners, time and staffing demands, regional variability in protocols, difficulty maintaining follow-up, and the practical complexity of safely delivering medicine sessions in group formats. Although group therapy may improve scalability by allowing more participants to receive care with fewer therapist hours, the literature suggests that group PaT remains operationally demanding and requires careful screening, staffing, safety monitoring, preparation, and integration procedures. Importantly, safety reporting in the included studies focused primarily on acute physiological or psychiatric adverse events, such as blood pressure changes, nausea, vomiting, headache, dizziness, suicidality, or serious adverse events. Few, if any, studies systematically assessed more nuanced or longer-term adverse experiences.

## Discussion

### Clinical impacts of group therapy with and without adjunctive psychedelics

There were notable differences in reported results between studies that only present one cohort, with all participants partaking in psychedelics, and studies that have comparison cohorts. The lack of significant differences in improvement between group therapy with and without adjunctive psychedelics raises an important question about the relative contributions of the psychedelic medicine, the therapeutic and group components, and their interaction. However, the absence of clear between-group differences on conventional clinical scales should not be interpreted as evidence that psychedelic experiences lack added value. Several included studies reported outcomes that extended beyond strict diagnostic categories, including social connectedness, meaning-making, spiritual wellbeing, psychological flexibility, and broader relational or existential change. These outcomes may be clinically meaningful even when they are not fully captured by symptom-based measures such as depression, anxiety, or PTSD scales.

There are some major barriers to answering this question. Firstly, the field of psychedelics has had a lack of studies with a comparator arm (44, 45). In particular with this type of medicine, there are noted difficulties with blinding where previous studies have reported high rates of participant unblinding (42). While this is especially true with studies comparing psychedelics to placebo, studies with another medication as a comparator (often stimulants) have also had a high rate of unblinding (34). Furthermore, preparation and integration therapy is now regarded as the gold standard in PaT and few research or clinical protocols are considered ethical without some degree of therapeutic intervention accompanying the psychedelic experience (46). This limits the possibilities of future studies comparing psychedelic-only interventions with little to no accompanying therapy. However, despite notable barriers, it would be possible to conduct well-designed trials for group PaT therapy compared to group therapy without PaT. Synthesis of these studies with good patient matching between cohorts would allow for investigation between the role that psychedelics play in group therapy.

A current understanding of the interaction between psychedelics and group therapy is to regard the utility of psychedelic intervention as a means to access neuroplasticity with the transformational behavior or personality altering work only achieved through concurrent therapy. However, in some studies in this review, authors often describe an “unlocking” of rigidity or potential for patients who may otherwise be resistant to therapy, suggesting that a second mechanism of the role of psychedelic intervention is to allow for a means to access therapy where previous lack of insight, interest, or potential may be barriers.

This second mechanism may be similar to what Cheek et al. (24) claimed in 1966 that

“LSD would appear to be a valuable tool for getting through' to the alcoholic, shaking him out of his narrowly focussed pattern of egocentric, resentful, guilt-ridden, alcoholic thinking.”

However, the mechanisms of action of psychedelics on the brain and body has also come to be a topic of immense discussion in recent years. It is known that many psychedelics and psychedelic-like medicines also have direct effects on the serotonergic and mesolimbic pathways (46). In addition, Lee et al. (47) conclude that it appears that

“psilocybin alters amygdala activity and glutamate levels in the medial prefrontal cortex, and increases functional connectivity and synaptic plasticity in the hippocampus.”

They also hypothesize that psilocybin's psychotropic therapeutic effects may be more broad than restricted to this neurotransmitter system alone.

Thirdly, it is known that broadening attention helps contribute toward a positive mood (48, 49) and the mood-enhancing effects of being in nature may be but one example of this. Subjective experiences of patients undergoing PaT readily note the interconnectedness between self and others, expanding toward oneness with the universe (50). Objective markers of this include mystical experience questionnaires where patients report “experience with the insight that all is one” or the “experience of unity with ultimate reality.”

The reported increased mindfulness noted by participants may also have contributed to improved clinical outcomes. These are also mechanisms of broadening attention which further contributes to improvements in mood in addition to its additive effects on the impact of social cohesion. Group PaT in contrast to individual PaT allows for natural peer to peer support during and after therapy which has been hypothesized to contribute to the ongoing integration process and sustained remission post-treatment (11). Previous studies have demonstrated the efficacy of peer groups in improving depression and other mental health disorders with similar effects when compared to gold standard treatments such as cognitive behavioral therapy (43).

These findings certainly support those studies in this review where despite a few hours of group therapy, significant improvement in psychiatric symptoms were reported. However, with these known pathways and theoreticized impacts, it remains unclear why psychedelic cohorts of therapy protocols do not result in more positive clinical and statistical significance when compared to group therapy-only cohorts when comparisons have been made available. Rather than viewing individual and group components as competing mechanisms, the newer studies suggest that hybrid models may be especially useful: individual sessions can provide screening, safety assessment, privacy, and personalized therapeutic containment, while group sessions can provide normalization, peer witnessing, interpersonal learning, and sustained integration.

### Individual and group therapy

Across the included studies, “group PaT” did not represent a single standardized intervention, but rather a family of models that varied by molecule, indication, degree of individual therapist contact, whether medicine administration occurred individually or simultaneously in groups, number of preparation and integration sessions, and whether the group was organized around a shared diagnosis, occupation, or identity. This heterogeneity limits direct comparison across studies but also illustrates the adaptability of group-based psychedelic care across clinical contexts.

The group therapy models presented in this review include named psycholytic models such as permissive group therapy (29) where patients spent months in regular group psychedelic sessions and therapy in a child-like inpatient environment. Although a model like this would not be ethical in today's research environment, more recent models include multi-week group therapy programs with two or more preparatory sessions before introduction of psychedelics, one or more psychedelic medicine sessions, and prompt integration therapy starting within 24 h of the psychedelic session. Some of these PaT protocols are described as traditional group therapy formats with adjunctive psychedelics or psychedelic-like substances (11, 25, 26), while others offer individual therapy “break outs” or a combination of individual and group preparation and integration session (17, 20). Lastly, there are PaT protocols offering individual therapy with group medicine sessions or vice versa group therapy with individual medicine sessions (17, 20). This last group was not discussed in our review as we were specifically interested in group participation throughout the various aspects of the protocol.

There are many benefits of group therapy for patients with mental health and or concurrent disorders compared to individual therapy at baseline including support through an identified peer group, the opportunity to point out affective states and transference processes between group members, and pragmatic benefits of reduced labor and costs. However, although many papers discussed the combination of group with individual therapy time, there were no papers that compared group PaT to individual PaT. It has been hypothesized that the group aspect of group PaT has additive benefits to the therapeutic process. Notably, Thomas et al. argues that the group cohesion that PaT participants endorsed may be attributable to the known pathways of psychedelics in promoting prosocial behaviors between study participants (40). In addition, the opportunity created by psychedelic medicines for neuroplasticity allows for potential reversal of maladaptive behavioral patterns through increased insight and practice (25). The social wellness findings are particularly important because they point to outcomes that extend beyond strict diagnostic categories. Improvements in belonging, interpersonal connection, spiritual connectedness, and general wellbeing suggest that group PaT may have transdiagnostic relevance, not only through symptom reduction but also through broader changes in relational and existential functioning. Therefore it may be the case that group PaT is not only pragmatically beneficial for its feasibility and cost effectiveness, it is important to the therapeutic outcomes of the intervention itself.

## Limitations and future directions

Limitations of this systematic review include the small number of high-quality comparative studies despite the broader number of papers identified. Although 30 papers met inclusion criteria, relatively few used randomized or controlled designs, and several reported overlapping cohorts or secondary analyses from the same parent study. In addition, there was significant heterogeneity among the studies including the inclusion of ketamine, a dissociative anesthetic, leading to difficulties in comparable outcomes. Many of the papers also predate current versions of the DSM-5 or ICD code which may create further inconsistencies in the inclusion criteria. Furthermore, the breadth of substance use disorders included in these studies is lacking with a primary focus on alcohol use disorders. The lack of long-term follow up in these studies also leave a lot to be determined regarding long-term efficacy and remission rates associated with treatment with group psychedelic assisted therapies. A key limitation of the current evidence base is the predominance of uncontrolled pre–post study designs. While many studies reported improvements in psychiatric symptoms and psychosocial outcomes, these findings cannot be interpreted as causal effects of the intervention. Improvements may reflect regression to the mean, placebo or expectancy effects, or the therapeutic impact of group-based support independent of the psychedelic intervention.

### Future directions

Future research would be important in exploring the comparison between individual and group PaT and the benefits and drawbacks of each type of therapy. In particular, an underexplored area in PaT includes the degree and influence of a participant's mystical experience and connection with clinical outcomes.

Future research studies should learn toward use of randomized controlled trials with some attempt at blinding and adequate power in order to glean statistically meaningful results that can then be correlated with clinical outcomes. In addition, one major limitation of this study is the heterogeneity of scales used to quantify results. If possible, scales should be standardized for this field of research to allow for comparable results between studies. Future studies should also broaden safety assessment beyond acute physiological and psychiatric adverse events to include longer-term existential, relational, spiritual, and integration-related difficulties which is especially important in group PaT.

Furthermore, in some studies, group therapy was used as a modality only in select components of the therapeutic process whereas in other studies, group therapy was used in all components. It will be important to elucidate where group therapy has the most value and to ask participants specifically about these experiences in order to further refine therapy protocols in the field. Here, the qualitative data aids in defining objective results from validated scales.

Given the lack of studies with follow-up for more than 6 months, it would be worthwhile noting which types of patients follow up and which do not. It would also be meaningful to ask patients what outcomes matter most to them and what their future needs are post group PaT. As with any therapy modality, it would be helpful in future studies to better define patient selection criteria and who may be the best candidates for group PaT.

Across studies, group formats varied greatly. This heterogeneity makes broad hypothesis testing difficult and means that pooled conclusions about “group PaT” may obscure clinically important variation. This context dependence is central to both the promise and scalability challenge of group PaT, suggesting that future research should identify adaptable principles and population-specific models rather than assuming a single gold-standard group protocol. Future research should also include qualitative, ethnographic, and community-based participatory approaches to better understand how group PaT functions across different cultural, relational, and geographic contexts. This is especially important for communities affected by medical racism and structural exclusion, where standard protocols may not generalize.

## Conclusions

This review suggests that group-based PAT is associated with improvements in psychiatric symptoms and psychosocial outcomes across a range of mental health conditions. However, given the predominance of uncontrolled study designs and heterogeneity in interventions and outcome measures, these findings should be interpreted with caution. While the group component may represent a potentially important aspect of these interventions, current evidence is insufficient to determine its specific contribution relative to individual therapy or other contextual factors. As such, the role of group-based approaches should be considered hypothesis-generating, rather than established. Overall, the certainty of evidence is low to moderate, and future research using controlled and comparative designs is needed to clarify the mechanisms and relative effectiveness of group-based PAT.

## Data Availability

The original contributions presented in the study are included in the article/Supplementary material, further inquiries can be directed to the corresponding author.
