# Supplementary material for: Group psychedelic-assisted therapy for mental health conditions: a mixed-methods systematic review
Source: Front Public Health. 2026 Oct 6;14:1788352. doi: 10.3389/fpubh.2026.1788352 (PMC13680196; doi:10.3389/fpubh.2026.1788352)
Supplement: Supplementary file 1 [file Table_1.docx]

**Appendix 1: Search strategy**

MEDLINE (Ovid)

| **#** | **Query** |
| --- | --- |
| 1 | (((Group or "cop" or community or communities or peer or peers) adj5 (therap* or psychotherap* or support or supports or program* or practice or “counsel*”)) or "Twelve step program*" or “12 step program*” or "Community of Practice*").mp. |
| 2 | Psychotherapy, Group/ |
| 3 | 1 or 2 |
| 4 | (Ketamine* or Psychedelic* or Hallucinogen* or Entheogen* or entactogen* or "MDMA" or "3,4-Methylenedioxymethamphetamine" or "lysergic acid diethylamide" or "LSD" or "5-MeO-DMT" or ayahuasca or iowaska or kamarampi or yage or huasca or psilocyb* or psilocib* or psilocibin* or psilocin* or "Ibogaine*" or "10-metoxyibogamine*" or Mescaline* or "N,N-dimethyltryptamine" or "peyote*").mp. |
| 5 | 3 and 4 |

APA PsycInfo (EBSCO)

| **#** | **Query** |
| --- | --- |
| S1 | DE "Encounter Group Therap*" OR DE "Therapeutic Community" OR DE "Support Group*" OR DE "Twelve Step Program*" OR DE “12 Step Program*” OR DE "Group Counsel*" OR DE "Group Psychotherap*" |
| S2 | TI ( (((Group or "cop" or community or communities or peer or peers) N5 (therap* or psychotherap* or support or supports or program* practice or counsel*)) or "Twelve step program*" or “12 step program*” or "Community of Practice*") ) OR AB ( (((Group or "cop" or community or communities or peer or peers) N5 (therap* or psychotherap* or support or supports or program or programs or practice or counsel*)) or "Twelve step program*" “12 step program*” or "Community of Practice*") ) OR KW ( (((Group or "cop" or community or communities or peer or peers or counsel*) N5 (therap* or psychotherap* or support* or program* or practice)) or "Twelve step program*" “12 step program*” or "Community of Practice*") ) |
| S3 | DE "Communities of Practice" |
| S4 | S1 OR S2 OR S3 |
| S5 | DE "Ketamine" OR DE "Lysergic Acid Diethylamide" OR DE "Mescaline" OR DE "Methylenedioxymethamphetamine" OR DE "Peyote" OR DE "Psilocybin" OR DE "Psychedelic Assisted Therapy" OR DE "Psychedelic Experiences" OR DE "Psychedelic Drugs" |
| S6 | TI ( (Ketamine* or Psychedelic* or Hallucinogen* or Entheogen* or entactogen* or "MDMA" or "3,4-Methylenedioxymethamphetamine" or "lysergic acid diethylamide" or "LSD" or "5-MeO-DMT" or Ayahuasca or iowaska or kamarampi or yage or huasca or psilocyb* or or psilocib* or psilocin* or "Ibogaine*" or "10-metoxyibogamine*" or Mescaline* or "N,N-dimethyltryptamine" or “peyote”) ) OR AB ( (Ketamine* or Psychedelic* or Hallucinogen* or Entheogen* or entactogen* or "MDMA" or "3,4-Methylenedioxymethamphetamine" or "lysergic acid diethylamide" or "LSD" or "5-MeO-DMT" or Ayahuasca or iowaska or kamarampi or yage or huasca or psilocyb* or or psilocib* or psilocin* or "Ibogaine*" or "10-metoxyibogamine*" or Mescaline* or "N,N-dimethyltryptamine" or “peyote”) ) OR KW ( (Ketamine* or Psychedelic* or Hallucinogen* or Entheogen* or entactogen* or "MDMA" or "3,4-Methylenedioxymethamphetamine" or "lysergic acid diethylamide" or "LSD" or "5-MeO-DMT" or Ayahuasca or iowaska or kamarampi or yage or huasca or psilocyb* or psilocin* or "Ibogaine*" or "10-metoxyibogamine*" or Mescaline* or "N,N-dimethyltryptamine" or “peyote”) ) |
| S7 | S5 OR S6 |
| S8 | S4 AND S7 |

Scopus

( TITLE-ABS-KEY ( ( ( *"Group"* OR *"groups"* OR *"cop"* OR *"community"* OR *"communities"* OR *"peer"* OR *"peers"* ) W/2 ( *"therapy"* OR *"therap*"* OR *"psychotherap*"* OR *"support"* OR *"supports"* OR *"program*"* OR *"practice"* OR *"counsel*"* ) ) OR *"Twelve step program*"* OR “12 step program*” OR *"Community of Practice*"* ) ) AND ( TITLE-ABS-KEY ( ( ( *"Ketamine*"* OR *"Psychedelic*"* OR *"Hallucinogen*"* OR *"Entheogen*"* OR *"entactogen*"* OR *{MDMA}* OR *"3,4-Methylenedioxymethamphetamine"* OR *"lysergic acid diethylamide"* OR *{LSD}* OR *"5-MeO-DMT"* OR *{Ayahuasca}* OR *"psilocyb*"* OR *"psilocib*"* OR *"psilocin*"* OR *"Ibogaine*"* OR *"10-metoxyibogamine*"* OR *"Mescaline*"* OR *"N,N-dimethyltryptamine"* OR *"peyote"*
