# Supplementary material for: Group psychedelic-assisted therapy for mental health conditions: a mixed-methods systematic review
Source: Front Public Health. 2026 Oct 6;14:1788352. doi: 10.3389/fpubh.2026.1788352 (PMC13680196; doi:10.3389/fpubh.2026.1788352)
Supplement: Supplementary file 2 [file Table_2.docx]

**Appendix 2: MMAT-Style Appraisal of 30 Psychedelic Group-Therapy Papers**

***Key: Y = criterion met; N = criterion not met; CT = cannot tell from available reporting. Criteria met counts are descriptive only; MMAT guidance discourages relying on a single pooled score.***

| **#** | **Paper** | **MMAT category applied** | **C1** | **C2** | **C3** | **C4** | **C5** | **Criteria met** | **Overall appraisal** | **Main rationale** |
| --- | --- | --- | --- | --- | --- | --- | --- | --- | --- | --- |
| **1** | **Agin-Liebes** | **Qualitative** | **Y** | **Y** | **Y** | **Y** | **CT** | **4/5** | **Moderate-High** | **Strong fit between question, microphenomenological interviews, and IPA analysis; findings are grounded in participant accounts. Reflexivity/researcher influence is less fully developed.** |
| **2** | **Agrawal 2023/2024 cancer MDD efficacy** | **Quantitative nonrandomized** | **CT** | **Y** | **Y** | **N** | **Y** | **3/5** | **Moderate** | **Prospective phase 2 open-label single-arm trial with validated outcomes and safety reporting; no control group, no randomization, and residual confounding remain major limitations.** |
| **3** | **Agrawal long-term follow-up** | **Quantitative nonrandomized** | **CT** | **Y** | **CT** | **N** | **Y** | **2-3/5** | **Moderate-Low** | **Valuable 2-year follow-up of the same cancer-MDD cohort, but still uncontrolled, small, and vulnerable to attrition/survivorship bias.** |
| **4** | **Beaussant qualitative acceptability** | **Qualitative** | **Y** | **Y** | **Y** | **Y** | **Y** | **5/5** | **High** | **Clear qualitative question, semi-structured exit interviews, template/thematic analysis, triangulation with clinical observations/quantitative findings, and explicit trustworthiness discussion.** |
| **5** | **Cheek** | **Quantitative descriptive / historical case series** | **CT** | **CT** | **CT** | **N** | **N** | **0-1/5** | **Low** | **Historical LSD alcoholism paper with limited modern reporting, unclear sampling/response procedures, and limited standardized measurement. Useful historically but weak by MMAT standards.** |
| **6** | **Chwelos** | **Quantitative descriptive / historical case series** | **CT** | **CT** | **CT** | **N** | **CT** | **0-1/5** | **Low** | **Describes LSD treatment in alcoholism, including difficult-prognosis patients, but has limited standardized outcomes, unclear representativeness, and older reporting standards.** |
| **7** | **Dames 2021** | **Mixed methods / program evaluation** | **Y** | **CT** | **CT** | **CT** | **CT** | **1-2/5** | **Moderate-Low** | **Program evaluation used mixed methods, validated symptom scales, and narrative feedback, but integration of qualitative and quantitative components is limited and design is uncontrolled quality improvement.** |
| **8** | **Flynn 2025** | **Quantitative nonrandomized / retrospective single-arm pre–post case series** | **CT** | **Y** | **Y** | **N** | **Y** | **3/5** | **Moderate-Low** | **This was a small retrospective single-arm repeated-measures case series of 16 self-selected first responders and frontline healthcare workers receiving a 6-week group KAP intervention. It used validated pre–post measures for anxiety, depression, and trauma symptoms and appropriate matched-pair analyses, including Wilcoxon signed-rank tests and effect sizes. However, there was no control group, participants were self-referred based on work-related stress, formal psychiatric diagnostic evaluation was not required, follow-up was limited to post-intervention, and confounding/expectancy effects could not be controlled.** |
| **9** | **Flynn 2025** | **Quantitative nonrandomized retrospective** | **CT** | **Y** | **CT** | **N** | **Y** | **2-3/5** | **Moderate-Low** | **Retrospective cohort using validated measures across timepoints; limited by self-referral, no control group, limited confounder handling, and small sample.** |
| **10** | **Lewis 2023 HOPE observations/impressions** | **Qualitative / descriptive qualitative** | **Y** | **Y** | **Y** | **Y** | **CT** | **4/5** | **Moderate-High** | **Qualitative survey and therapist-process observations are well matched to the aim of informing group-format protocol design; less formal than a full qualitative interview study.** |
| **11** | **Lewis 2025 MBSR + psilocybin RCT** | **Quantitative randomized controlled trial** | **Y** | **CT** | **Y** | **N** | **Y** | **3-4/5** | **High-Moderate** | **Strongest design in the set: randomized active-comparator trial with validated outcomes and follow-up. Limitations include small n, homogeneous population, limited blinding, and unequal intervention intensity.** |
| **12** | **Oehen 2022 Swiss MDMA/LSD model** | **Quantitative descriptive / clinical-practice case series** | **CT** | **N** | **CT** | **CT** | **CT** | **0-1/5** | **Low-Moderate** | **Real-world clinical-practice report with 50 licensed patients and safety observations; heterogeneous diagnoses and treatments, no control group, and clinical-judgment outcomes limit appraisal.** |
| **13** | **Robison 2022 eating-disorder G-KAP** | **Mixed methods case series** | **CT** | **CT** | **CT** | **CT** | **CT** | **1-2/5** | **Low-Moderate** | **Uses validated symptom measures and clinical/participant observations, but n = 5, no control group, and limited qualitative integration.** |
| **14** | **Robison 2023/2024 frontline healthcare workers KAP** | **Mixed methods case series** | **CT** | **CT** | **CT** | **CT** | **CT** | **1-2/5** | **Low-Moderate** | **Validated PTSD/depression/anxiety measures plus feedback; limited by n = 10, no comparator, case-series design, and limited integration of qualitative and quantitative strands.** |
| **15** | **Rush / ATOP Takiwasi** | **Mixed methods observational cohort** | **Y** | **Y** | **Y** | **Y** | **CT** | **4/5** | **Moderate-High** | **Strong naturalistic mixed-methods evaluation: quantitative outcomes over time plus qualitative reports in a complex therapeutic-community setting. Main limitation is lack of control and inability to isolate ayahuasca from broader program.** |
| **16** | **Sevelius / Kindred** | **Mixed methods pilot** | **Y** | **Y** | **Y** | **Y** | **Y** | **5/5** | **High for feasibility/pilot aims** | **Explicit convergent mixed-methods design, parallel quantitative and qualitative data, integration during interpretation, clear feasibility aims, and transparent pilot limitations. Not powered for efficacy.** |
| **17** | **Smart controlled lysergide alcoholism study** | **Quantitative randomized / controlled trial** | **CT** | **CT** | **Y** | **CT** | **Y** | **2-3/5** | **Moderate for historical era** | **Stronger than many LSD-era papers because it used comparison groups, but reporting of randomization, allocation concealment/blinding, and baseline comparability is limited by contemporary standards.** |
| **18** | **Gasser 1995 Swiss psycholytic therapy** | **Quantitative descriptive / follow-up** | **CT** | **CT** | **CT** | **CT** | **CT** | **Not fully appraisable** | **Low / unclear** | **Uploaded paper is image-only with limited extractable methods; appears to report Swiss psycholytic therapy follow-up, but full MMAT appraisal is limited by inaccessible/unclear methods detail.** |
| **19** | **Giovanetti Yaqui ayahuasca program** | **Quantitative descriptive / observational pilot** | **Y** | **CT** | **Y** | **CT** | **Y** | **3/5** | **Moderate-Low** | **Clear community-based observational pilot using symptom measures; limited by no control, small sample, short-term follow-up, and context-specific sampling.** |
| **20** | **Hausner & Dolezal LSD group/individual psychotherapy** | **Quantitative nonrandomized historical study** | **CT** | **CT** | **CT** | **N** | **CT** | **0-1/5** | **Low** | **Includes multiple groups/conditions, but allocation, confounding, outcome validity, and completeness are unclear by modern standards.** |
| **21** | **Jensen alcoholism mental-hospital program** | **Quantitative descriptive / historical program report** | **CT** | **N** | **CT** | **CT** | **CT** | **0-1/5** | **Low** | **Large program description but limited standardized outcome measurement, unclear sampling and follow-up, and heavy confounding from multi-component treatment.** |
| **22** | **Lewis 2023 HOPE clinical outcomes** | **Quantitative nonrandomized pilot trial** | **Y** | **Y** | **Y** | **N** | **Y** | **4/5** | **Moderate-High** | **Prospective open-label pilot with validated depression outcome, safety reporting, and 26-week follow-up; major limitation is single-arm design with n = 12 and no comparator.** |
| **23** | **Spencer permissive LSD group therapy** | **Qualitative/descriptive clinical case series** | **Y** | **CT** | **CT** | **Y** | **CT** | **2/5** | **Low-Moderate historically; Low by MMAT** | **Rich clinical description of LSD group therapy context, but lacks formal qualitative sampling, analytic method, or standardized outcome procedures.** |
| **24** | **Thomas ayahuasca-assisted therapy for addiction/stress** | **Mixed methods prospective observational** | **Y** | **CT** | **Y** | **CT** | **CT** | **2-3/5** | **Moderate** | **Pre/post and 6-month data plus qualitative participant experience; limited by small n, no control, self-selection, and incomplete integration.** |
| **25** | **Tsang 1 CoP with/without KAT pilot** | **Quantitative nonrandomized retrospective** | **CT** | **Y** | **CT** | **N** | **Y** | **2-3/5** | **Moderate-Low** | **Retrospective secondary analysis with validated PHQ-9, GAD-7, and PCL-5, and comparison of CoP-only vs CoP+ketamine; limited by self-selection into ketamine and confounding.** |
| **26** | **Tsang 2 RTT-KaT safety review** | **Quantitative descriptive** | **Y** | **CT** | **Y** | **Y** | **Y** | **4/5** | **Moderate-High for safety question** | **Larger retrospective chart review focused on safety/tolerability across 128 participants and 448 sessions; representativeness is unclear, but measurements and analysis are appropriate for adverse-event description.** |
| **27** | **Tsang 3 firefighter qualitative paper** | **Qualitative case study** | **Y** | **Y** | **Y** | **Y** | **CT** | **4/5** | **Moderate-High for qualitative aim** | **Clear qualitative aim focused on firefighter experiences beyond symptom reduction; interviews and follow-up survey are relevant, but reflexivity/researcher influence is not fully developed.** |
| **28** | **Sokolik & Hausner** | **Descriptive clinical report** | **CT** | **CT** | **CT** | **CT** | **CT** | **Not fully appraisable** | **Low / unclear** | **Historical image-only paper with limited accessible methods detail. It appears clinically descriptive rather than a formally structured empirical study.** |
| **29** | **Tsang 4 firefighter prospective observational case series** | **Mixed methods / observational case series** | **Y** | **CT** | **Y** | **CT** | **CT** | **2-3/5** | **Moderate-Low** | **Prospective observational firefighter case series with validated measures and qualitative themes; limited by n = 8, no control group, and selection bias.** |
| **30** | **Wopfner Klinik Sudhang** | **Quantitative descriptive / program-development report** | **CT** | **CT** | **CT** | **CT** | **CT** | **1/5 or not fully appraisable for efficacy** | **Low-Moderate for program description; Low for efficacy** | **Useful description of standardized psilocybin-assisted group psychotherapy program and safety experience, but limited formal outcome data and limited information for efficacy appraisal.** |
